# Mycobacterial α-glucans hijack Dectin-1 to facilitate intracellular bacterial survival

**DOI:** 10.1126/sciimmunol.adw0732

**Published:** 2026-01-09

**Authors:** Shota Torigoe, Sumayah Salie, Roanne Keeton, Beren Aylan, Ben J. Appelmelk, David L. Williams, Douglas W. Lowman, Toshihiko Sugiki, Sohkichi Matsumoto, Akira Kawano, Satoru Mizuno, Kazuhiro Matsuo, Jonas N. Søndergaard, James B. Wing, Maxine A. Höft, Romey Shoesmith, Mthawelanga Ndengane, Anna K. Coussens, Janet A. Willment, Maximiliano G. Gutierrez, Jennifer Claire Hoving, Sho Yamasaki, Gordon D. Brown

**Affiliations:** 1 Laboratory of Molecular Immunology, Immunology Frontier Research Center, Osaka University, Osaka, Japan; 2 Leprosy Research Center, National Institute of Infectious Diseases, Japan Institute for Health Security, Tokyo, Japan; 3 Management Department of Biosafety, Laboratory Animal, and Pathogen Bank, National Institute of Infectious Diseases, Japan Institute for Health Security, Tokyo, Japan; 4 CMM AFRICA Medical Mycology Research Unit, Institute of Infectious Diseases and Molecular Medicine (IDM), University of Cape Town, Cape Town 7925, South Africa; 5 Host-pathogen interactions in tuberculosis laboratory, The Francis Crick Institute, London, UK; 6 Medical Microbiology and Infection Control, Amsterdam University Medical Centers, Amsterdam, The Netherlands; 7 Department of Surgery, James H. Quillen College of Medicine, East Tennessee State University, Johnson City, TN, United States; 8 Laboratory of Molecular Biophysics, Institute for Protein Research, Osaka University, Osaka, Japan; 9 Department of Bacteriology, Graduate School of Medical and Dental Science, Niigata University, Niigata, Japan; 10 Department of Microbiology and Molecular Genetics, Mie University Graduate School of Medicine, Mie, Japan; 11 Institute for Vaccine Research and Development, Hokkaido University, Hokkaido, Japan; 12 Human Single Cell Immunology Team, Center for Infectious Disease Education and Research (CiDER), Osaka University, Suita, Japan; 13 Human Single Cell Immunology, Immunology Frontier Research Center, Osaka University, Osaka, Japan; 14 Centre for Infectious Diseases Research in Africa, Institute of Infectious Disease and Molecular Medicine, University of Cape Town, South Africa; 15 Department of Pathology, University of Cape Town, South Africa; 16 Infectious Diseases and Immune Defense Division, Walter and Eliza Hall Institute of Medical Research, Australia; 17 Department of Molecular Immunology, Research Institute for Microbial Diseases, Osaka University, Osaka, Japan; 18 Laboratory of Molecular Immunology, Center for Infectious Disease Education and Research, Osaka University (CiDER), Osaka, Japan; 19 Division of Molecular Immunology, Medical Mycology Research Center, Chiba University, Chiba, Japan; 20 Division of Molecular Design, Research Center for Systems Immunology, Medical Institute of Bioregulation, Kyushu University, Fukuoka, Japan; 21 Medical Research Council Centre for Medical Mycology, University of Exeter, Exeter, United Kingdom

## Abstract

Mycobacteria possess a cell envelope that can act as a shield against host defense. This study shows that mycobacteria survive in host macrophages by targeting the innate host receptor Dectin-1 through a non-canonical ligand. Compared to WT mice, Dectin-1-deficient mice were more resistant to infection to mycobacteria. Indeed, Dectin-1-deficient mice presented with substantially reduced bacterial burdens, inflammatory cytokines and infiltrating myeloid cells, such as neutrophils and macrophages. Intracellular survival of these bacteria was reduced in macrophages derived from Dectin-1-deficient mice compared with those from WT mice. Cellular characterization of mycobacterial infected macrophages indicated that the presence of Dectin-1 altered phagosomal maturation and association with markers of autophagy. Activity-based purification and nuclear magnetic resonance spectrometry identified branched α-glucan as the Dectin-1 mycobacterial ligand. This branched glucan was essential for activating Dectin-1. These results show that mycobacterial α-glucan targets Dectin-1 to facilitate intracellular bacterial survival.

## Introduction

Despite over a century of research, tuberculosis remains one of the deadliest bacterial infections in humans, resulting in over one-and-a-half million deaths each year. Infection with *Mycobacterium tuberculosis* (MTB) occurs following inhalation of bacteria into the lung and the subsequent uptake of these microorganisms by phagocytic cells. Phagocytic cells detect MTB through innate pattern recognition receptors (PRRs), which trigger intracellular signalling cascades that induce a range of cellular and immunological responses. These important innate immune interactions influence the development of the disease, but MTB has also evolved multiple strategies to overcome these defenses, manipulating host cells to facilitate intracellular survival and replication (1, 2).

The C-type lectin receptors (CLRs) are one family of PRRs that are able to recognize glycan structures expressed on the surface of most microorganisms (3). Dectin-1 (gene symbol *Clec7a*), is one of the best studied CLRs and is broadly expressed on myeloid cells, in both human and mouse, inducing intracellular signalling that triggers numerous responses, including actin-mediated phagocytosis, the production of inflammatory mediators and enhances microbial killing and antigen presentation (3). Dectin-1 also regulates autophagy, in part through activation of mTOR (3–7).

Dectin-1 has been extensively studied in the context of fungal pathogenesis through its ability to recognize β-glucans. However, Dectin-1 is involved in immunity to other pathogens, including mycobacteria (8). Indeed, we and others have previously demonstrated that mycobacterial recognition by Dectin-1 induces several cellular responses, including the production of inflammatory cytokines and the respiratory burst (9–16). These *in vitro* studies suggested that Dectin-1 was involved in mounting protective immune responses. Yet in vivo loss of Dectin-1 led to reductions (~0.5 log) in the pulmonary bacillary burdens of mice infected with the laboratory strain of MTB H37Rv, suggesting that Dectin-1 could be driving non-protective responses (17). In this study, we have discovered that mycobacterial α-glucan is recognized by Dectin-1, an interaction which facilitates intracellular bacterial survival and drives susceptibility to infection.

## Results

### Dectin-1 drives susceptibility to mycobacterial infection in vivo

**Figure 1:**
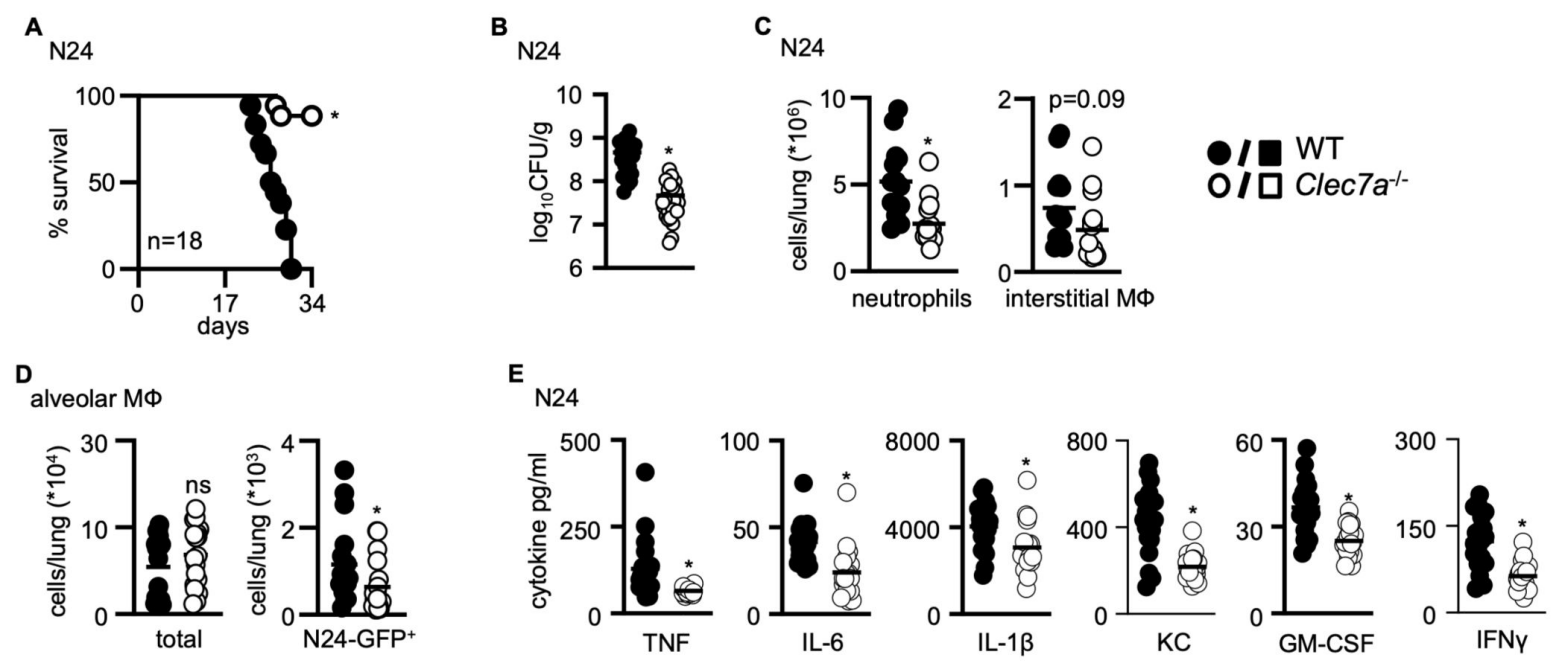
Dectin-1 deficient mice are resistant to mycobacterial infection (**A**) Survival of WT (n = 18) and *Clec7a*^–/–^ (n = 18) 129Sv mice after intranasal (i.n.) infection with the clinical strain *M. tuberculosis* (MTB) N24. Pooled data from two experiments (day 1 loading control - 400, 687 CFU). Pulmonary bacterial burdens (CFU, bar indicate average; n = 18) (**B**) and cellular composition (n =18) (**C**) in the lungs of 129Sv WT and *Clec7a*^*–/–*^ mice 21 days after infection with MTB N24. Neutrophils were defined as CD11b^+^Ly6G^+^ and interstitial (Int.) macrophages (MФ) as CD11b^+^CD11c^-^SiglecF^-^F4/80^+^. (**D**) Total (left) and number of GFP^+^ alveolar macrophages (right) in the lungs of mice 14 days after i.n. infection with 400-600 CFU of MTB N24-GFP (n = 18). (**E**) Concentrations of inflammatory cytokines in lung homogenates at day 21 following infection with MTB N24 in WT and *Clec7a*^–/–^ mice, as determined by Luminex cytokine profiling (n = 8). All data shown are pooled data from at least two independent experiments, with bar charts showing the mean ± SEM. Student’s *t*-tests were used for statistical analyses, except for (**A**), where survival was compared using the log-rank test. *p < 0.05.

To examine the function of Dectin-1 during infection with clinical strains of MTB, wild–type (WT) and *Clec7a*^–/–^ mice were infected by intranasal (i.n.) administration of 400-600 CFU of *M. tuberculosis* N24 (18). Notably, survival of *Clec7a*^–/–^ mice were prolonged, compared to WT animals (Fig. 1A). To rule out a bacterial strain dependent phenotype (19, 20), we examined survival of mice after infection with other strains of MTB, including Beijing and Erdman. For infection with either strain, the loss of Dectin-1 was protective such that increased survival was observed in the *Clec7a*^–/–^ mice (fig. S1A). We could also show increased survival of *Clec7a*^–/–^ mice infected with a higher dose of H37Rv than examined previously (17) (fig. S1A).

To gain insight into the underlying differences in pathology following infection, we analyzed WT and *Clec7a*^–/–^ mice 21 days after infection with MTB N24. This time point was selected as it occurred prior to the onset of mortality in WT animals (Fig. 1A). While there were no obvious differences in the gross pathology in the infected lungs at this time point (fig. S1B), we found that *Clec7a*^– /–^ mice had more than a log fold reduction in pulmonary bacterial burdens, indicating that there was improved control of infection in the Dectin-1 deficient animals (Fig. 1B). We also observed reduced numbers of pulmonary neutrophils and interstitial monocytes/macrophages in the lungs of infected *Clec7a*^–/–^ mice by flow cytometry (Fig. 1C). Similar differences in pulmonary inflammatory cell infiltration were observed using CyTOF in WT and *Clec7a*^–/–^ mice infected with MTB Erdman (figs. S1C-E). Using a GFP-expressing MTB N24, we detected fewer macrophages harboring MTB in the lungs of infected *Clec7a*^–/–^ mice at day 14 post infection (Fig. 1D). There was no difference between the numbers of neutrophils harboring MTB at this time point (fig. S1F). Consistent with the reduced numbers of inflammatory cells, there were reduced levels of inflammatory cytokines and chemokines in the lungs of infected *Clec7a*^–/–^ mice (Fig. 1E). There were no alterations in the number of T cells recruited to the lungs of infected *Clec7a*^–/–^ mice (figs. S1C-E), nor differences in T cell recall responses to MTB antigen stimulation (fig. S1G). Taken together, our data show that deficiency of Dectin-1 is protective during pulmonary infection with MTB, and that this likely stems from alterations of innate immune responses.

### Dectin-1 promotes mycobacterial survival by altering intracellular trafficking

**Figure 2:**
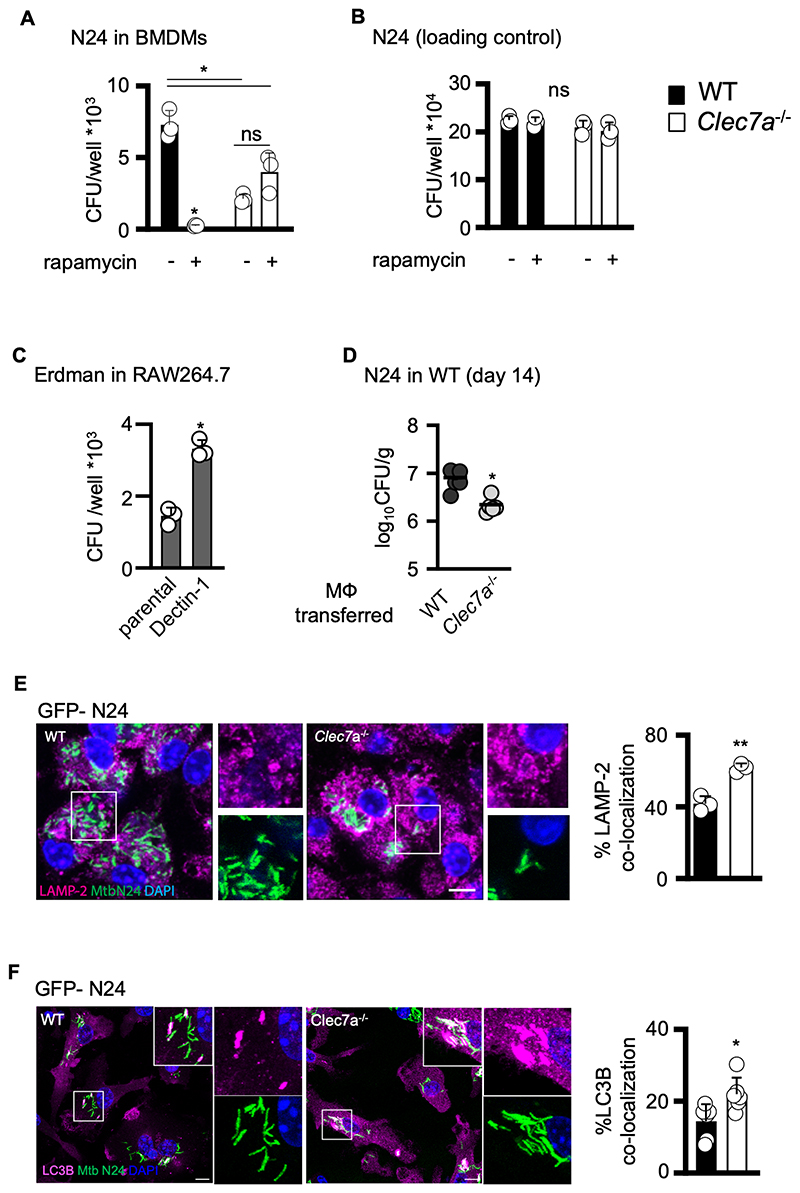
Dectin-1 promotes intracellular mycobacterial survival (**A**) CFU counts at day 6 following infection of 129Sv WT and *Clec7a*^–/–^ BMDMs with MTB N24, in the presence or absence of 1µM rapamycin, as indicated. Data show mean ± SD from one representative experiment performed in triplicate. (**B**) CFU counts at day 0 (loading control) following infection of 129Sv WT and *Clec7a*^–/–^ BMDMs with MTB N24, in the presence or absence of 1 µM rapamycin, as indicated. Data show mean ± SD from one representative experiment performed in triplicate. (**C**) CFU counts at day 6 following infection of null or Dectin-1-overexpressing RAW264.7 macrophages with MTB Erdman, as indicated. Data show mean ± SD of one representative experiment performed in triplicate. (**D**) Pulmonary bacterial burdens (CFU, bar indicate average; n = 5) in the lungs of 129Sv WT mice 14 days after intratracheal transfer of 1*10^5^ WT or *Clec7a*^*–/–*^ BMDMs, as indicated. Data from a single experiment. (**E**) Confocal microscopy images (left) and quantitation (right, data show mean ± SD) of LAMP-2 (red) co-localization in 129Sv WT and *Clec7a*^–/–^ BMDMs 24 hr following infection with MTB N24-GFP. DNA was stained with DAPI (blue). Scale bar = 10µm. (**F**) Confocal microscopy images (left) and quantification (right, data show mean ± SD) of LC3B (red) in 129Sv WT and *Clec7a*^–/–^ BMDMs 48 hr following infection with MTB N24-GFP. DNA was stained with DAPI (blue). Scale bar = 10µm. (**E, F**) Data representative of 2 experimental repeats with least 25 fields of view analyzed in each experiment, with three technical repeats conducted in each experiment. All data are representative of at least two independent experimental repeats. Student’s *t*-test (**D, E, F**) or ANOVA (**A, B, C**) were used for statistical analyses. *p < 0.05.

Macrophages are key phagocytic cells during the development of tuberculosis (2, 21), so we explored the outcome of infection with MTB N24 in WT and *Clec7a*^–/–^ macrophages *in vitro*. Remarkably, we found that loss of Dectin-1 led to reduced bacterial burdens in infected bone-marrow derived macrophages (BMDMs; Fig. 2A), even when the macrophages were treated with IFNγ (fig. S2A). There was no difference in mycobacterial uptake between Clec7a^-/-^ and WT macrophages (Fig. 2B), nor differences in TNFα production following infection with intact mycobacteria, as previously reported (fig. S2B) (9, 10). Similar results were observed following infection with MTB Beijing (fig. S2C) Notably, using RAW264.7 macrophages that express negligible levels of Dectin-1 (22), we could show that overexpression of Dectin-1 led to higher bacterial burdens (Fig. 2C). Importantly, bacterial burdens could be reduced during infection *in vivo* by intratracheal transfer of knockout macrophages into WT mice (Fig. 2D)

As intracellular control of mycobacteria in macrophages is dependent on phagosome maturation (23, 24), we used microscopy to explore the fate of internalized MTB N24. We did not detect a difference in phagosomal maturation using lysotracker red (fig. S2D), presumably due to loss of staining following phagosomal escape by MTB (25) By contrast, we detected higher association of the lysosomal marker, LAMP-2, with internalized GFP-N24 MTB in *Clec7a*^–/–^ macrophages (Fig. 2E). Notably, we also detected higher association of the autophagy markers, LC3B and p62, with intracellular MTB in the knockout cells (Fig. 2F and figs. S2. E and F). LC3 processing and p62 accumulation were observed by protein immunoblot following infection of the cells with MTB (fig. S2F)(26, 27), although this technique was not sufficiently sensitive to detect differences between the WT and *Clec7a*^–/–^ macrophages that we observed through imaging individual cells. Nevertheless, these results indicate that Dectin-1 promotes intracellular mycobacterial survival by influencing phagosome maturation and activation of the autophagy pathway.

Dectin-1 signalling activates mTOR, thus supressing autophagy (4–7). Indeed, we found that the addition of an mTOR inhibitor, rapamycin, had a profound effect on WT macrophages, enabling them to effectively control intracellular MTB (Fig. 2, A and B). By contrast, rapamycin had little effect on the control of intracellular growth of MTB in *Clec7a*^–/–^ macrophages (Fig. 2, A and B). Rapamycin itself had no direct anti-mycobacterial activity (fig. S2G).

### Dectin-1 recognizes mycobacterial α-glucan

**Figure 3:**
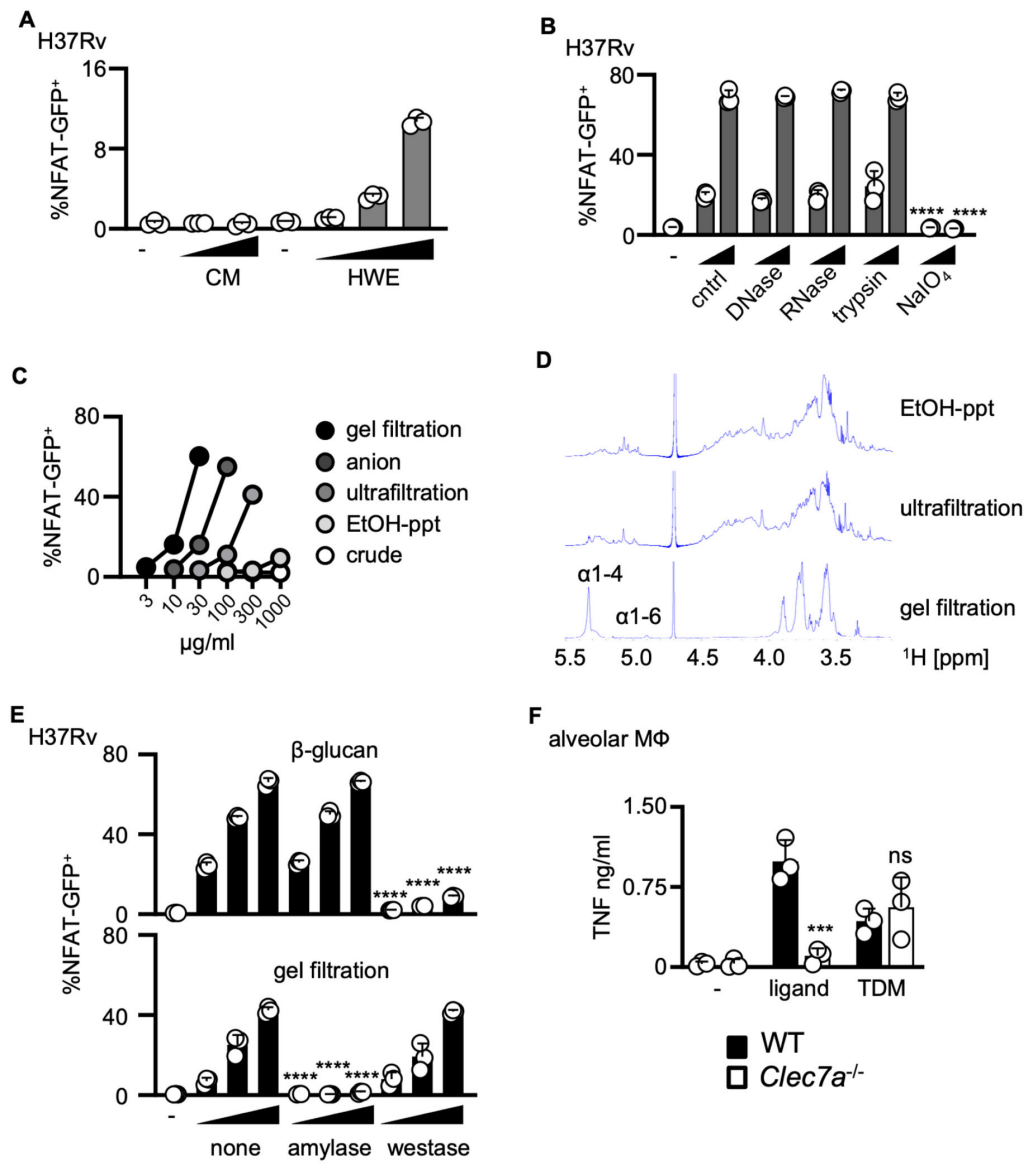
Dectin-1 recognizes mycobacterial α-glucan (**A**) NFAT-GFP reporter cells expressing murine Dectin-1 were stimulated with plate-coated chloroform methanol (C:M) extracts or hot water extracts (HWE) (0.1, 0.3, and 1 mg/mL) isolated from MTB H37Rv for 18 hr and analyzed by flow cytometry. Unstimulated cells (-) are shown as a control. Data show mean ± SD from triplicate assays and are representative of at least two independent experiments (**B**) Human NFAT-GFP reporter cells were stimulated with untreated (cntrl), DNase-treated, RNase-treated, trypsin-treated or NaIO_4_-treated HWE for 18 hr and analyzed for GFP expression by flow cytometry. Data show mean ± SD from triplicate assays and are representative of at least two independent experiments (**C**) Reporter cells expressing human Dectin-1 were stimulated with crude, ethanol precipitated (EtOH-ppt), ultrafiltration, anion exchange and gel filtration purified components for 18 hr and analyzed for GFP expression by flow cytometry. Data show a triplicate assay and are representative of at least two independent experiments (**D**) ^1^H-NMR spectrum of mycobacterial HWE components following EtOH-ppt, ultrafiltration and gel filtration purification. (**E**) Reporter cells expressing human Dectin-1 were stimulated with gel filtration purified ligand (3, 10, 30 µg/ml) or β-glucan (curdlan; 1, 3, 10 µg/ml) treated with or without amylase or westase for 18 hr and analyzed for GFP expression by flow cytometry. Data show mean ± SD from triplicate assays and are representative of at least two independent experiments. (**F**) C57BL6/J WT or *Clec7a*^–/–^ alveolar macrophages were stimulated for 24 hr with 100 µg/ml of purified MTB ligand and trehalose di-mycolate (TDM, a ligand for the related receptor MINCLE) (0.1 µg/well) and cytokine production analyzed by ELISA. Data (A, B, E, F) show mean ± SD from one representative experiment performed in triplicate. One-way ANOVA (A, B, **E, F**) were used for statistical analyses. **** *p* < 0.001, **** *p* < 0.0001.

Mycobacteria do not possess β-glucans, a known ligand of Dectin-1. To identify the mycobacterial ligand recognized by Dectin-1, we screened mycobacterial extracts using nuclear factor of activated T cells (NFAT)-green fluorescent protein (GFP) reporter cells expressing murine Dectin-1 (28). Murine Dectin-1 expressing reporter cells reacted to hot water extract (HWE)-derived hydrophilic components in a dose-dependent manner but did not react to the chloroform/methanol (C:M) extracted lipophilic fraction (Fig. 3A). Similar results were obtained using extracts from MTB N24 and with reporter cells expressing human Dectin-1 (figs. S3A & B). To determine the nature of the Dectin-1 ligand, mycobacterial HWE was treated with enzymes digesting DNA, RNA, protein and with NaIO_4_ to degrade saccharides. We found the Dectin-1 ligand to be sensitive to NaIO_4_, but not to DNase, RNase, or trypsin (Fig. 3B), suggesting that the ligand is a saccharide. We also found that the ligand to be shed into the growth media following the culture of MTB (fig. S3C).

To deterimine the molecular identity of the ligand, we purified the active component (fig. S3D). We first enriched the ligand using 85% ethanol precipitation (EtOH-ppt) and ultrafiltration (MW50000), detecting the presence of ligand activity using Dectin-1 reporter cells (Fig. 3C). Active fractions containing saccharides that exhibited Dectin-1 specific activity were combined and separated using anion exchange and then gel filtration column chromatography (figs. S3E and S3F). This approach yielded a purified Dectin-1 ligand-containing extract, with a molecular weight of 80,000–150,000 (fig. S3F). Following purification, the specific activity was increased (Fig. 3C). Using the Dectin-1 reporter cells, we showed that recognition of this purified ligand extract by Dectin-1 involved amino acid residues known to be required for recognition of β-glucans (fig. S3G) (29). We also demonstrated that a soluble Fc-Dectin-1 protein, but not the related Fc-CLEC12A (30), bound to the ligand using a solid phase immunoassay (fig. S3H).

We then used nuclear magnetic resonance (NMR) to determine the structure of the Dectin-1 ligand in the purified extract. The ^1^H-NMR spectrum revealed proton signals of α-1,4 and α-1,6 linked glucose moieties, which were enriched as compared to less purified material (Fig. 3D). Iodine staining of the purified material revealed a brown color similar to that seen with glycogen (a branched α-1,4 glucan), but not amylose (a linear α-1,4 glucan), suggesting that the mycobacterial ligand was composed of α-1,4 glucan backbone with α-1,6 linked branches (fig. S3I). Indeed, we could demonstrate increased purification of branched α-1,4 glucan using a commercial kit to measure glycogen content (fig. S3J). We also confirmed the nature of the mycobacterial ligand by demonstrating loss of activity following treatment with amylase, which digests α-1,4 linkages, but not westase, which digests β1,3-glucans (Fig. 3E). Moreover, using enzymatic digestion, we could demonstrate that the ligand did not contain any other major mycobacterial polysaccharides including alpha mannan, arabinomannan, arabinogalactan, lipomannan or lipoarabinoman (fig. S3K). Notably, the purified α-glucan could induce proinflammatory cellular responses in isolated alveolar macrophages in a Dectin-1 dependent manner (Fig. 3F), as previously described for purified β-glucans (3), but was not recognized by other C-type lectins including DC-SIGN and Mincle (fig. S3L).

To further demonstrate a direct interaction of Dectin-1 with the purified mycobacterial ligand, we carried out Saturation Transfer Difference (STD)-NMR experiments using soluble Fc-Dectin-1 protein (fig S3M). As before, the ^1^H NMR analysis of the purified ligand showed the characteristic H1, H3, H2-4, H5-6, which occurs in α-glucan (31), and H2’-H6b’ showed the presence of the non-reducing end. Positive signals were detected in the STD spectrum, indicating a direct interaction between Dectin-1 and purified ligand (fig. S3N). The STD spectrum showed that the highest degree of saturation was attained at protons H2-4 and H5-6, but not at the non-reducing end. Thus, together these results show that Dectin-1 recognizes a branched mycobacterial α-glucan.

### Vaccine strains and other mycobacterial species also express the Dectin-1 ligand

**Figure 4:**
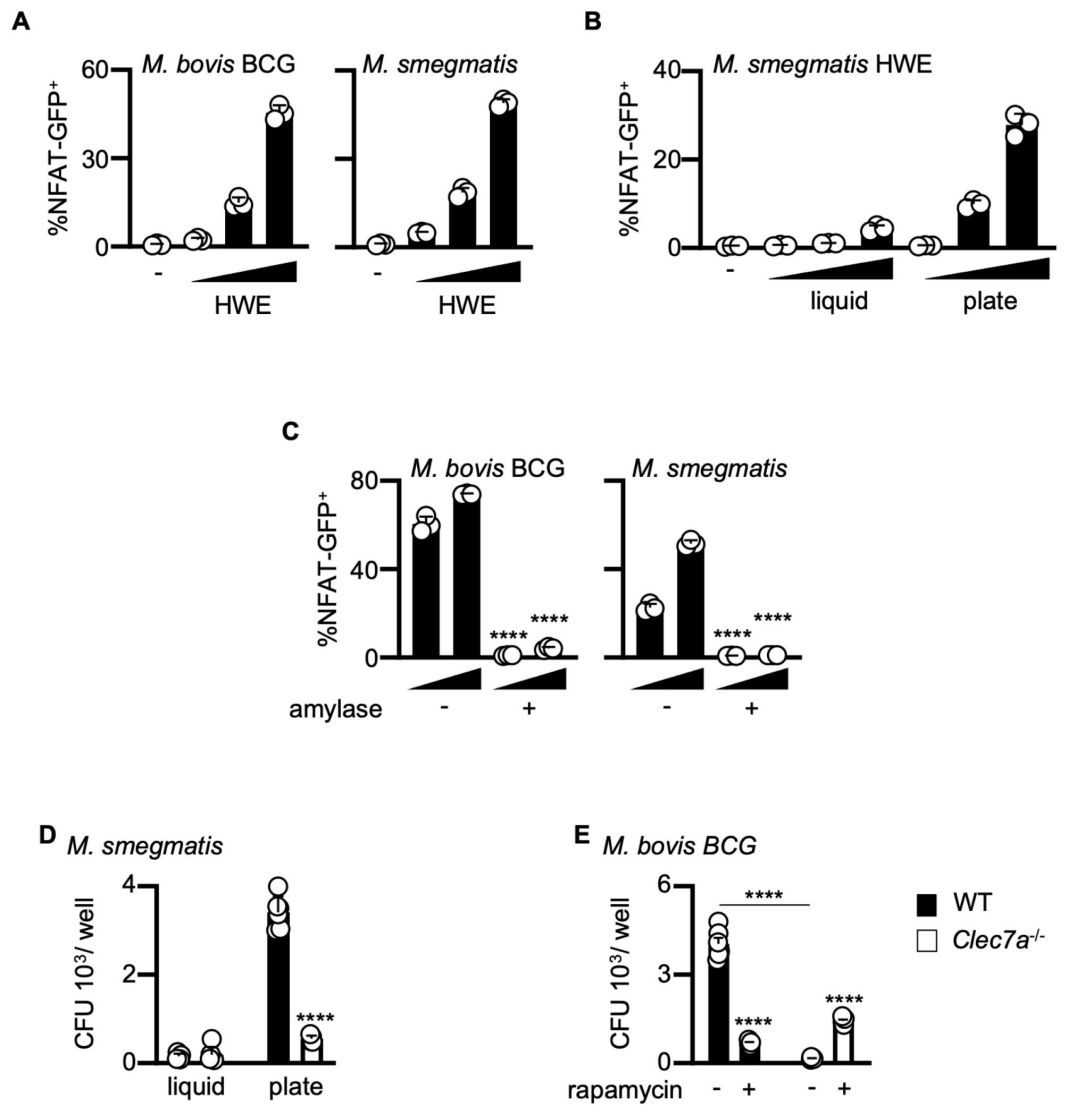
Vaccine strains and other mycobacterial species also express the Dectin-1 ligand (**A**) NFAT-GFP reporter cells expressing human Dectin-1 were stimulated for 18 hr with *M. smegmatis* or *M. bovis* BCG derived HWE (0.03, 0.1, and 0.3 mg/mL). GFP expression was analyzed using flow cytometry. (**B**) Reporter cells expressing human Dectin-1 were stimulated for 18 hr with HWE extracted from *M. smegmatis* cultured in liquid media or harvested directly from agar plates. GFP expression was analyzed using flow cytometry. (**C**) NFAT-GFP reporter cells were stimulated for 18 hr with non- or amylase-treated HWE from *M. smegmatis* or *M. bovis* BCG and analyzed for GFP expression, by flow cytometry. (**D**) CFU counts at day 6 following infection of 129Sv WT and *Clec7a*^–/–^ BMDMs with *M. smegmatis*, harvested from liquid media or directly from agar plates. (**E**) CFU counts at day 6 following infection of 129Sv WT and *Clec7a*^–/–^ BMDMs with *M. bovis* BCG, in the presence or absence of 1µM rapamycin, as indicated. (**A - C**) Data are the mean ± SD of triplicate assays and representative of two independent experiments. (**D-E**) Pooled data from two independent experiments showing mean ± SEM. One-way ANOVA (**C-E**) were used for statistical analyses. **** *p* < 0.0001.

α-Glucans are conserved in mycobacteria (32), so we determined if Dectin-1 recognizes these carbohydrates in vaccine strains and other mycobacterial species. Indeed, we found that HWEs from *M. bovis* BCG and *M. smegmatis* could stimulate Dectin-1 reporter cells (Fig. 4A). Notably, the Dectin-1 ligand was only detected when extracts were prepared from cells harvested directly from agar plates, as opposed to those harvested following liquid culture (Fig. 4B and fig. S4A). Through enzymatic digestion with amylase, purification, and NMR analyses, as before, we demonstrated that Dectin-1 was recognizing branched α-glucans in these species (Fig. 4C and fig. S4B). We found that the intracellular survival of these virulent mycobacteria in WT macrophages was increased when harvested directly from agar plates (Fig. 4D and figs. S4, A and D). Moreover, we found that this survival advantage was abolished when these bacteria infected *Clec7a*^–/–^ macrophages (Figs.4, D and E, and figs. S4, A and D). As we found for MTB, inhibiting the mTOR pathway with rapamycin in WT macrophages restored the ability of these phagocytes to rapidly control intracellular mycobacteria (Fig. 4E and fig. S4D). Inhibiting the mTOR pathway with another inhibitor, Torin, also restored the ability of WT macrophages to control intracellular mycobacteria (fig. S4E). Thus, recognition of avirulent mycobacteria by Dectin-1 promotes mycobacterial survival within macrophages.

### Recognition of branched α-glucans by Dectin-1 promotes mycobacterial survival in macrophages

**Figure 5:**
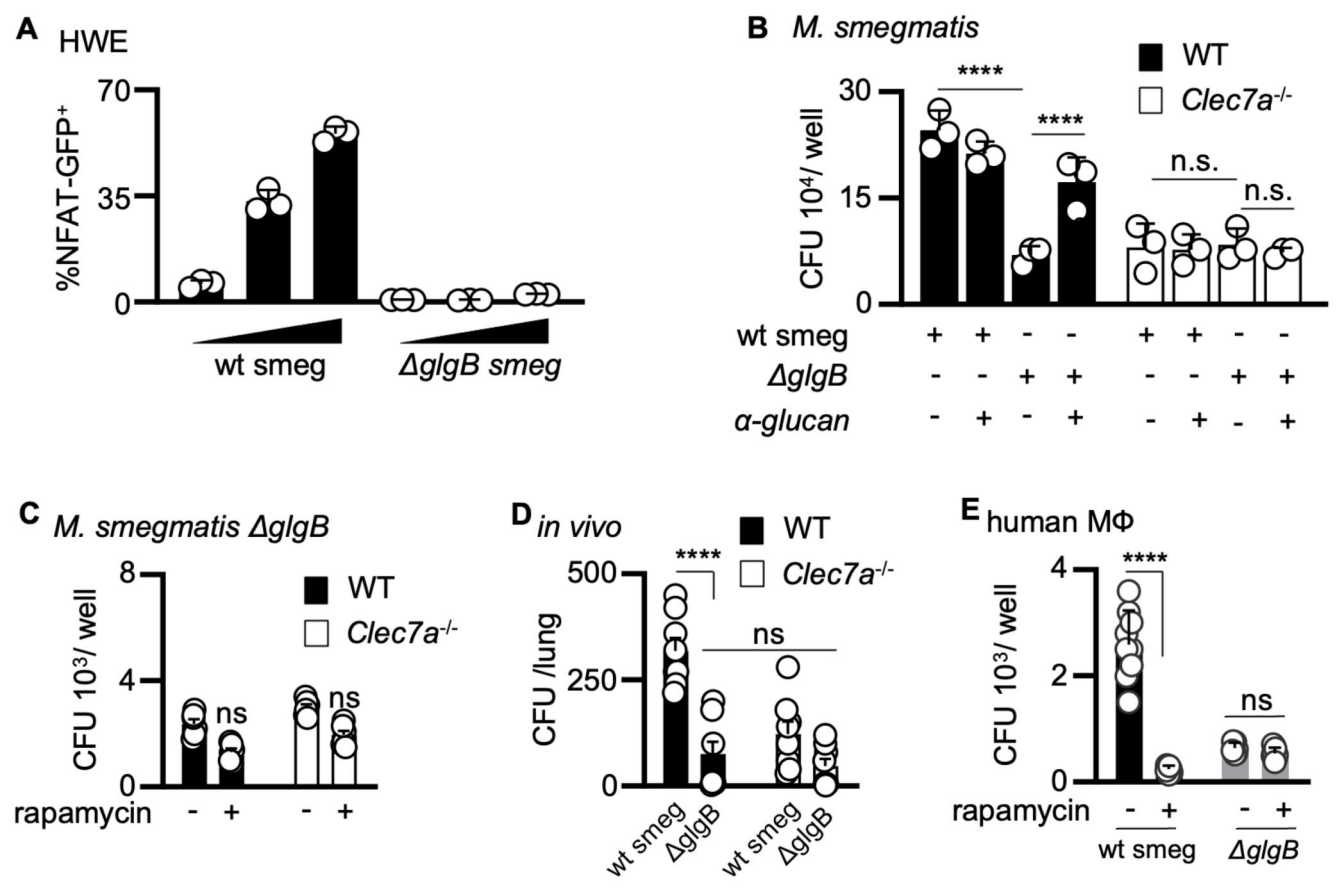
Recognition of branched α-glucans by Dectin-1 promotes mycobacterial survival in macrophages (**A**) NFAT-GFP reporter cells expressing human Dectin-1 were stimulated for 18 hr with HWE extracted from *M. smegmatis* WT or the Δ*glgB* mutant and analyzed for GFP expression by flow cytometry. Data are the mean ± SD of triplicates and representative of two independent experiments. (**B**) CFU counts at day 2 following infection of C57BL6/J WT and *Clec7a*^–/–^ BMDMs with *M. smegmatis* WT (wt smeg) or the Δ*glgB* mutant, as indicated, in the presence or absence of purified mycobacterial α-glucan (500 µg/ml). Data from one experiment showing mean ± SD. (**C**) CFU counts at day 6 following infection of 129Sv WT and *Clec7a*^–/–^ BMDMs with the Δ*glgB* mutant, in the presence or absence of 1µM rapamycin, as indicated. (**D**) Pulmonary bacterial burdens (CFU, bar indicate average) in the lungs of 129Sv WT and Clec7a^–/–^ mice (n=8) 1 day after infection with *M. smegmatis* WT (wt smeg) or the Δ*glgB* mutant. (**C**-**D**) Data pooled from two independent experiments showing mean ± SEM. (**E**) CFU counts at day 6 following infection of human peripheral blood derived macrophages with *M. smegmatis* WT (wt smeg) or the Δ*glgB* mutant, as indicated. Data are the mean ± SD of triplicates pooled from cells isolated from three donors. One-way ANOVA was used for statistical analyses. **** *p* < 0.0001.

To formally demonstrate that recognition of branched α-glucans by Dectin-1 promotes the intracellular survival of mycobacteria, we made use of an *M. smegmatis* mutant lacking the α1-6 branching enzyme (Δ*glgB)* (31). We could not generate MTB mutants lacking this enzyme, as they are non-viable (33). As reported previously (31), iodine staining of the *M. smegmatis* Δ*glgB* colonies yields a dark-blue color, due to the linear α1-4-glucans excessively present in mutant (fig. S5A) (31). Importantly, HWEs prepared from *M. smegmatis* Δ*glgB* cells were unable to stimulate Dectin-1 reporter cells (Fig. 5A). Furthermore, the survival of the Δ*glgB* mutant was attenuated in WT macrophages but unaltered in *Clec7a*^–/–^ cells (Fig. 5B). Complementation of the *M. smegmatis* Δ*glgB* strain with *glgB* restored the ability of the mycobacteria to survive longer within WT but not *Clec7a*^–/–^ cells (figs. S5, C and D). Remarkably, the addition of soluble α-glucan to the Δ*glgB* strain during infection was sufficient to enhance mycobacterial survival in WT, but not Dectin-1-deficient, macrophages (Fig. 5B). The addition of soluble α-glucan with WT *M. smegmatis* did not affect its intracellular survival in either macrophage cell type (Fig. 5B). Moreover, inhibition of mTOR with rapamycin did not alter the intracellular survival of the Δ*glgB* mutant in either WT or *Clec7a*^–/–^macrophages (Fig. 5C). We could also demonstrate that the presence of α-glucans prolonged *M. smegmatis* bacterial survival following infection in WT but not *Clec7a*^–/–^, mice (Fig. 5D and fig. S5B), although the mice were still able to clear these bacteria at later timepoints. Finally, we confirmed that α-glucans increased *M. smegmatis* survival in human monocyte derived-macrophages, and that this effect could be overcome following mTOR inhibition with rapamycin (Fig. 5E). Thus, these data show that recognition of branched α-glucan by Dectin-1 promotes intracellular mycobacterial survival in macrophages.

## Discussion

Dectin-1 is an archetypical pattern recognition receptor best studied in the context of fungal infections, where it plays a key role in protective antifungal immunity (30). Several studies have also implicated this C-type lectin in immunity to other pathogen classes, including mycobacteria, although definitive *in vivo* physiological functions for this receptor have not been clearly established in this context. Here we show that in contrast to fungi, the activities of Dectin-1 promote susceptibility to mycobacterial infection. Indeed, loss of Dectin-1 enhanced the ability of mice to resist infection with MTB, with *Clec7a*^-/-^ animals having reduced bacterial burdens, lower levels of inflammatory cytokines and chemokines, and a reduced influx of key inflammatory cells including neutrophils and macrophages/monocytes. We could show that this phenotype was independent of the bacterial and mouse strain examined and stemmed from Dectin-1’s recognition of mycobacterial α-glucan, which facilitated intracellular bacterial survival.

MTB possesses multiple mechanisms to ensure its survival following ingestion by macrophages. MTB alters phagosomal processing pathways, and it damages host phagosomal membranes that escape into the cytosol, actively evading both canonical (xenophagy) and non-canonical (LC3-associated phagocytosis, LAP) autophagy (26, 27, 34). In the absence of Dectin-1, we observed increased association of MTB with LAMP-2 and the autophagy markers, LC3 and p62, suggesting enhanced targeting to the xenophagy pathway. How the autophagy pathway is activated during MTB infection is not completely understood but involves both canonical and non-canonical targeting of MTB to LC3^+^ compartments (35). Whereas Dectin-1 can promote LC3B recruitment to fungal phagosomes (36), this receptor also induces activation of mTOR (4–6). mTOR blocks canonical autophagy pathways (37), and inhibition of this enzyme is known to be protective during mycobacterial infection (23). We found that inhibition of mTOR in WT, but not Dectin-1-deficient, cells decreased intracellular mycobacterial survival. Whereas inhibition of mTOR has been linked to mitochondrial function and susceptibility to mycobacterial infection in zebrafish models and THP-1 cells (38), neither express Dectin-1 (39, 40). However, this mechanism may explain the slightly increased mycobacterial survival we observed in some experiments in the Dectin-1-deficient cells following mTOR inhibition.

The role of autophagy as a protective antimycobacterial cellular immune response has been controversial (41). However, two recent studies have provided key insights into the mechanisms by which canonical and non-canonical autophagy controls phagosomal and cytosolic MTB (26, 27). We found that mTOR also regulates the intracellular control of non-pathogenic mycobacterial species, including *M. smegmatis*, which are retained within phagosomes and do not localize in the cytoplasm (42). Thus, it is unlikely that the autophagy machinery targets cytosolic bacteria in the processes described here. Indeed, mTOR-dependent autophagy components, including ATG7 and ATG14, are critical for restricting intracellular mycobacterial growth through control of fusion of mycobacterial phagosomes with lysosomes (27). It is important to emphasize that the role of autophagy in MTB control is still unclear, as it has been implicated in lysosomal targeting and membrane repair (34). Moreover, the signals that trigger LC3/Atg8 conjugation to MTB phagosomes are still unknown, likely involving complex pathways in which mTOR is only a part. Further studies are required to determine the precise pathways underlying the mechanisms we have detected here. Moreover, while our data strongly suggest that the phenotype we observe is due to alteration in macrophage functions, generation of a conditional Clec7a^-/-^ mouse would enable a more detailed dissection of the individual cellular functions of this receptor in antimycobacterial immunity.

α-Glucan is a known virulence factor present in the mycobacterial capsule, previously implicated in directing cellular uptake through complement receptor 3 (CR3), modulating DC responses (in part through recognition by another C-type lectin DC-SIGN (43)) and required for virulence in mouse models (44). Our data show that branched mycobacterial α-glucan is recognized by Dectin-1, and no other CLRs, triggering responses that promote mycobacterial survival within macrophages. Unexpectedly, we found that expression of this carbohydrate by avirulent *M. smegmatis*, also prolonged intracellular survival of the bacteria in a Dectin-1-dependent manner. However, this phenotype was only evident when the bacteria were cultured under conditions that promoted high levels of α-glucan production; reduced levels of α-glucan in the capsule of *M. bovis* BCG have been linked to the presence of detergents in liquid culture media (45).

How Dectin-1 recognizes any polysaccharide is still unclear. Lacking classical sugar-binding motifs, several aromatic amino acids flanking a groove on the surface of this receptor are known to be essential for recognition of β-glucans (29). These residues were also required for recognition of the branched mycobacterial α-glucans. Our data show that Dectin-1 recognizes branched, and not linear, mycobacterial α-glucans. Indeed, Dectin-1 does not recognize linear α-glucans (46), which is further evidenced by the inability of this CLR to recognize the Δ*glgB* mutant of *M. smegmatis*, in which these linear polysaccharides are still present (31). The mycobacterial α-glucan is also different from the linear α-glucan reported recently in fungi (47). This fungal α-glucan is recognized by other CLRs, including MINCLE, and (unlike the branched mycobacterial α-glucans) is unable to induce inflammatory responses in macrophages (47). The higher affinity of Dectin-1 for branched versus linear β-glucans (48) is suggestive of the mechanism underlying ability of this receptor to recognize and respond to branched mycobacterial α1,4 glucans.

What is the relevance of our observations for human disease? We found that human Dectin-1 also recognizes mycobacterial α-glucan and could show that these carbohydrates enhanced bacterial survival in human macrophages. Notably, we found that human macrophages can control intracellular survival of mycobacteria following treatment with rapamycin, as has been observed in other studies (49). These data indicate that recognition of mycobacterial α-glucan by Dectin-1 also promotes mycobacterial survival in human cells, and that the functions of this receptor promote disease susceptibility. Of interest, polymorphisms of Dectin-1 have been identified which adversely affect immunity to fungal pathogens, such as the Y238X polymorphism in which homozygous individuals are essentially Dectin-1-deficient (50). These polymorphisms are present at high frequencies (50) suggestive of retention through evolutionary pressure. We are currently exploring the hypothesis that such polymorphisms of Dectin-1 may have co-evolutionarily arisen in human populations (51) to provide protection against mycobacterial infection.

## Limitation / Caveats

Despite these findings, our study has several limitations. First, we relied on global Clec7a^–/–^ mice on two genetic backgrounds and therefore cannot fully exclude contributions of Dectin-1 in non-myeloid compartments; conditional deletion of Clec7a in defined myeloid subsets will be required to definitively assign the phenotype to macrophage-intrinsic functions. Second, our conclusions regarding altered phagosome maturation and autophagy are based mainly on imaging readouts and pharmacological inhibition of mTOR, and we did not genetically dissect individual components of the autophagy machinery or mTOR signalling cascade, nor visualize LC3^+^ compartments in infected lungs in vivo. Third, because the α1,6-branching enzyme GlgB is essential in M. tuberculosis, the formal genetic demonstration that branched α-glucan is required for Dectin-1–dependent survival was performed in *M. smegmatis*; although the biochemical features of the α-glucans are conserved, the relative contribution of this pathway may vary among *M. tuberculosis* lineages and clinical isolates. Finally, we did not assess the impact of naturally occurring human Dectin-1 polymorphisms or evaluate mTOR inhibition as a host-directed therapy in vivo. Addressing these points in future studies will refine our understanding of how Dectin-1 and mycobacterial α-glucans shape host–pathogen interactions during tuberculosis and may inform the rational targeting of this pathway.

## Materials and Methods

### Study design

#### Predefined study components

No formal statistical power analysis was performed before data collection. Sample sizes and group numbers for in vivo and in vitro experiments were determined on the basis of previous experience with similar tuberculosis infection and macrophage assays, and are reported in the figure legends. Data collection for each experiment was stopped once the pre-specified number of independent biological replicates had been completed. For in vivo infection studies, humane endpoints (including 15–20% body weight loss, respiratory distress, and moribund state) were defined a priori and used as criteria for euthanasia. All technically valid measurements that met predefined quality criteria (e.g. successful infection, absence of contamination or technical failure) were included in the analysis, and no data points were excluded as outliers on the basis of post hoc inspection. The primary endpoints were survival time to humane endpoint, pulmonary bacterial burdens, cytokine and chemokine concentrations in lung homogenates, frequencies and numbers of immune cell subsets by flow or mass cytometry, intracellular bacterial CFU in macrophage cultures, and fluorescence-based readouts from reporter cells and imaging assays.

#### Rationale and overall design

This study was designed to determine how Dectin-1 recognition of mycobacterial α-glucans regulates intracellular survival of mycobacteria and the resulting host immune responses during tuberculosis infection. We combined in vivo infection models in WT and Clec7a^−/−^ mice challenged with WT or mutant mycobacterial strains, with in vitro infections of murine and human macrophages and Dectin-1 reporter cells exposed to purified carbohydrate fractions and blocking reagents. Outcomes included host survival, bacterial burdens, lung pathology, cytokine and chemokine production, phagocyte activation, and microbial uptake, which were quantified using CFU enumeration, histology, flow and mass cytometry, microscopy and imaging, reporter readouts, and gene-expression analyses.

#### Randomization

For in vivo experiments, WT and Clec7a^–/–^ mice were co-housed and then randomly assigned to experimental groups (e.g. different infection strains or treatments) before infection. Infection doses were validated by CFU enumeration in separate animals at 18–24 hr post infection. In vitro experiments (macrophage infections, reporter cell assays, and imaging) were performed using parallel cultures or wells that were allocated to conditions in a non-systematic manner within each plate or experiment; no additional formal randomization procedure was applied.

#### Blinding

In vivo infection studies were not blinded: investigators were aware of the mouse genotype and treatment group during infection, monitoring, and endpoint analyses. In vitro experiments and imaging analyses were also conducted without blinding to experimental conditions, as the primary outcome measures (CFU counts, flow cytometric frequencies, and automated fluorescence intensity quantification) are objective, instrument-based readouts. No allocation concealment procedures were used.

#### Replication

Unless otherwise stated in the figure legends, all experiments were independently repeated at least twice. In vivo infection experiments were performed in at least two independent cohorts of mice per condition, with group sizes indicated in the figure legends. In vitro infection assays with murine BMDMs, RAW264.7 cells, and human monocyte-derived macrophages, as well as reporter-cell and imaging assays, were performed in at least two independent biological replicate experiments; within each experiment, multiple technical replicates (wells or fields of view) were analyzed for each condition. Replicate numbers (biological and technical) for individual experiments are specified in the corresponding figure legends.

Reporting of animal experiments in this study is consistent with the ARRIVE guidelines

### Animals

*Clec7a*^–/–^ mice on a 129Sv background were generated by homologous recombination as described previously (52) or on a C57BL6/J background through CRISPR-Cas9 (Fig. S6A-C). Aged and sex matched WT control animals were obtained from in-house colonies (129Sv, Cape Town) or from Clea, Japan (C57BL6/J). All mice were bred and maintained under specific-pathogen-free conditions and provided with standard laboratory food and water ad libitum. All experiments were conducted according to the guidelines for animal care approved by the Research Institute for Microbial Diseases, Osaka University, Research Institute of Tuberculosis, Tokyo, Japan BCG Laboratory or by the Animal Research Ethics Committee of the Health Sciences Faculty, University of Cape Town (Reference number: 018/018 & 022/025).

### Mycobacteria

*Mycobacterium tuberculosis* clinical strains N24, N24-GFP, Erdman, Beijing and H37Rv were grown in Middlebrook 7H9 broth (Difco™ BD Becton) supplemented with 10% Middlebrook oleic acid albumin dextrose catalase (OADC) enrichment (BBL™ Becton Dickinson Microbiology systems) and 0.05% Tween 80 (Sigma). Cultures were incubated at 37°C with continuous rolling until in mid log growth phase. Mycobacterial culture aliquots were stored at -80°C following the addition of 10% (v/v) glycerol (Sigma). Mycobacterial concentrations (colony forming units (CFU/ml)) were determined through serial dilution and plating on Middlebrook 7H10 agar (Difco™ BD Becton).

*M. bovis* BCG, *M. smegmatis* mc^2^155 and *M. smegmatis* Δ*glgB* were grown in Middlebrook 7H9 broth supplemented with 10% ADC enrichment and 0.05% Tween 80 and stored in aliquots at −80°C. *GlgB* complemented strains were generated using the pYM301 vector as described (53). For experiments, bacteria were prepared by thawing a frozen aliquot and diluting to the required concentration for infection. For plate cultures, strains were grown on 7H10 agar supplemented with OADC. Bacteria were harvested by scraping, washed, and diluted to the required concentration for infection/analyses.

### Preparation of human monocyte-derived macrophages

Buffy coats were obtained from anonymous blood donors through the Western Cape Blood Service, Cape Town, South Africa. Acquisition of human blood samples was approved by the Faculty of Health Sciences Human Research Ethics of the University of Cape Town (HREC #317/2016). Peripheral blood mononuclear cells (PBMCs) were isolated from buffy coats by density gradient centrifugation using Lymphoprep (Axis Shield), washed three times in PBS (Ca^++^ and Mg^+^ free), and cryopreserved at ≈20x10^6^ cells/ml in RPMI/20% FSC/5% DMSO. PBMC, in batches of three donors per experiment, were gently thawed resuspending in cold RPMI 1640 (always supplemented with 1 mM sodium pyruvate) and 2 mM L-glutamine (all from Sigma). CD14^+^ monocytes were isolated from PBMCs using CD14^+^ magnetic bead separation (Miltenyi), following manufacturer’s protocol with the modification of using 12 μl beads/10^7^ PBMC. Isolated monocytes were differentiated into macrophages by culturing at 2x10^6^ cells/3 ml in RPMI 1640 supplemented with 5 ng/ml GM-CSF (Miltenyi) and 10% human AB serum (Merck) for 7 days at 37°C/5% CO_2_. Following differentiation, monocyte-derived macrophages (MDMs) were washed with PBS and detached with two rounds of Accutase (Sigma) incubation at at 37°C/5% CO_2_ and washed in PBS. MDMs were then plated at 1x10^5^ cells/96-well in 200 µl RPMI 1640 (supplemented with 5% human AB serum) and rested at at 37°C/5% CO_2_ for one hour before infection.

### In vitro analyses

2B4-NFAT-GFP reporter cells expressing human or murine Dectin-1, murine Dectin-1 W221A variant, murine Dectin-1 H223A variant or murine Dectin-1 W228Y variant were prepared as described previously (28). All receptors were expressed at equivalent levels at the cell surface (Fig. S6D), following staining with isotype control or anti-Dectin-1 antibody (2A11; 1 µg/ml) and analyzed by flow cytometry. Reporter cells were suspended by 10% FCS supplemented RPMI1640 media at 300,000 cells/ml and 100 µl of the cell suspension was dispended into a 96-well plate (Corning). The cells were stimulated at at 37°C/5% CO_2_ for 16–20 hr as indicated in the figure legends. After stimulation, the cells were harvested and GFP expression of the cells was analyzed using flow cytometry (Gallios, Beckman Coulter). Mycobacterial components, purified α-glucan, and curdlan (InvivoGen) were prepared in double distilled water (10 mg/ml). For enzyme treatments, active components were treated with amylase (Sigma-Aldrich), westase (TAKARA Bio), mannosidase (New England Biolabs), arabinosidase (Megazyme) or galactosidase (Megazyme) in citrate buffer (pH 4.5) for 16-20 hr at 37ºC.

To collect murine alveolar macrophages, 1 ml of 1 mM EDTA-PBS was administered to the lung through the trachea of mice and washed repeatedly with round 24 G needle attached to a 1.5 ml syringe. Collected cells were washed with 2% FCS RPMI1640 media once and then suspended in 10% FCS RPMI1640 at 100000 cells/ml. 100 µl of the cells were dispensed into a 96 well plate. Murine alveolar macrophages stimulated with purified α-glucan (100 µg/ml) or trehalose dimycolate (0.1 µg/well) (Sigma-Aldrich) for 24 hr and TNF in supernatant was measured by ELISA (BD Bioscience).

For infection assays, murine bone-marrow derived macrophages (BMDM, prepared using standard methodology involving M-CSF (22)) or human MDM, were infected with the mycobacterial strain of interest at a multiplicity of infection (MOI) of 2:1 (mycobacteria:macrophage) for 4 hours at 37°/ 5% CO_2_. Thereafter, the cells were washed twice with PBS to remove extracellular bacilli and incubated at 37°C/ 5% CO_2_. In some experiments, 1μM rapamycin (Sigma-Aldrich) or 0.1µg/ml Torin-1 (Sigma-Aldrich) or 10 ng/ml IFNγ (Biolegend) was added after the washing step and maintained for the rest of the experiment. At the specified time points (days 0, 1 & 6), cells were lysed with cold sterile water and lysates plated on Middlebrook 7H10 agar (Difco™ BD Becton) plates supplemented with 10% OADC (v/v) (BBL™ Beckton Dickinson Microbiology systems) to enumerate surviving bacilli. Day 0 plates were lysed immediately after the washing step, to check for equal loading of the cells.

For RAW264.7 infection assays, null and Dectin-1 overexpressing RAW264.7 cells were infected with the mycobacterial strain of interest at a multiplicity of infection (MOI) of 2:1 (mycobacteria:macrophage) for 4 hours, washed and then incubated at 37ºC with 5% CO2. After 2 days, cells were lysed and CFU counts determined, as detailed above.

### Infection of human monocyte-derived macrophages

*M. smegmatis* mc^2^-155 and *M. smegmatis* Δ*glgb* were prepared from plated cultures and used to infect the human monocyte-derived macrophages. A MOI of 2:1 (mycobacteria:macrophage) was used and macrophages were incubated for 1 hr at 37°C with 5% CO_2_. Thereafter, the cells were washed twice with PBS to remove extracellular bacilli and incubated at 37°C with 5% CO_2_. All wells were always checked (day 0 CFU) for equal bacterial loading. In some experiments, 1 μM rapamycin (Sigma-Aldrich) was added after the washing step and maintained for the rest of the experiment. 24 hr post infection, cells were harvested with PBS containing 2 mM EDTA incubated at 37°C and any cells still adhering were gently scraped from the well. The lysates were plated on Middlebrook 7H10 agar (Difco™ BD Becton) plates supplemented with 10% OADC (v/v) (BBL™ Beckton Dickinson Microbiology systems) to enumerate surviving bacilli.

### *In vivo* infections and analyses

All WT and Clec7a^–/–^ mice were co-housed for at least 10 days prior to infection. Animals were randomly assigned to control and treatment groups, and the studies were not blinded. For survival experiments, mice were culled when they reached defined clinical endpoints including 15% (MTB erdman infection) or 20% (all other MTB strains), had difficulty breathing and/ or became moribund. Frozen aliquots of *Mycobacterium tuberculosis* clinical strains N24, N24-GFP, or Beijing, *M. smegmatis* mc^2^-155 or *M. smegmatis* Δ*glgb* were thawed at room temperature and clumps were disrupted by the addition of glass beads (Sigma) and vortexing for 30 seconds. The inoculum for *Mycobacterium tuberculosis* N24, N24-GFP and Beijing was prepared from liquid culture, and mice were challenged intranasally with doses resulting in 400-600 cfu/lung. In some experiments, 1*10^5^ WT or *Clec7a*^*-/-*^ BMDMs, prepared as described above, were administered intratracheally 24 hr before infection. For infections with *Mycobacterium tuberculosis* Erdman, mice were challenged with ~1300 cfu/lung via aerosol. For infections with *M. smegmatis* mc^2^-155 and / or *M. smegmatis* Δ*glgb*, inocula were prepared directly from culture plates and mice were infected with ±10 000 CFU/lung. Infection doses were validated by collecting lungs 18-24 hours post infection, and processed and plated for CFU counts as detailed below. Mice were euthanized when having lost 20% of their starting body weight and/ or when mice displayed signs of distress, including difficulty in breathing, hypo/hyperactivity, hunched posture and arching and pilo-erection.

To determine bacterial burdens, lungs were collected aseptically from infected mice, at the time points indicated in the text, homogenised and plated onto Middlebrook 7H10 agar (Difco™ BD Becton) supplemented with 10% ADC or OADC (v/v) (BBL™ Beckton Dickinson). For microscopy, the left lung lobe was excised from the infected animal, fixed with 10% formalin, cut to 2-5 μm thickness, mounted on APES (3-aminopropyltriethoxysilane, 99%) and stained with hemotoxylin and eosin. To determine tissue cytokine levels, lung homogenates were centrifuged to remove debris and supernatants stored at −80°C. Cytokine levels were measured using the Bio-Plex Pro Mouse 23-Plex kit (Bio-Rad) and read on a Bio-plex 200 system (Bio-Rad), according to the manufacturer’s instructions. Cytokine levels of tissue homogenates were normalized to sample protein concentrations.

### Flow cytometry

To obtain single cell suspensions for flow cytometry, whole lungs were cut into pieces and digested in DMEM for 90 minutes at 37°C with 50 units/ml Collagenase Type I (Gibco Life Technologies) and 13 μg/ml DNase I (Sigma-Aldrich). Digested lung tissue was passed through a 70μm cell strainer (Falcon), and the cells recovered by centrifugation at 4°C. Following lysis of red blood cells (156 mM NH_4_Cl, 0.127 mM EDTA, and 11.9 mM NaHCO_3_; Merck, Germany), cells were collected by centrifugation, resuspended in FACS media (DMEM (Gibco Life Technologies Limited, UK) containing 10% heat-inactivated Fetal Bovine Serum (iFBS) (Gibco Life Technologies Limited, USA)) and passed through a 40 μm cell strainer.

Lung single-cell suspensions in FACS buffer (PBS + 1% bovine serum albumin (BSA) (Rosche) and 0.1% NaN_3_ (Merck,)), were stained with the myeloid flow cytometry antibody cocktail. The myeloid panel included the following antibodies: APC rat anti-mouse Ly6G (clone: 1A8, BD Pharmingen, 1:100), Alexa Fluor® 700 hamster anti-mouse CD11c (clone: HL3, BD Pharmingen, 1:100), V450 rat anti-mouse CD11b (clone M1/70, BD Horizon, 1:100), APC-Cy 7™ rat anti-mouse SiglecF (clone: E50-2440, BD Pharmingen, 1:50), BV510 rat anti-mouse CD45 (clone: 30-F11, BD Horizon, 1:100) and PE/Cy7 anti-mouse F4/80 (clone: BM8, Biolegend, 1:100). A blocking agent was added to the cocktail to prevent non-specific binding of the antibodies: mouse BD Fc block™ rat anti-mouse CD16/CD32 antibody (clone:2.4G2, BD Pharmingen, 1:100). Cells were washed to remove excess unbound antibodies, centrifuged, and resuspended in 2% paraformaldehyde fixation buffer (v/v in PBS, Sigma-Aldrich, USA) before acquisition. Data acquisition was performed using an LSR Fortessa™ (BD Immunocytometry Systems, San Jose, CA, USA) and BD FACS Diva software (v6.0). Flowjo software (v10.0.7) (Treestar, Ashland, OR, USA) was used for post-acquisition analysis cell population determination. For the current study, neutophils were defined as CD11b^+^Ly6G^+^, inflammatory monocytes as CD11b^+^CD11c-SiglecF-F4/80^+^ and alveolar macrophages as CD11b^int^CD11c^+^SiglecF^+^F4/80^+^. Gating strategies for in vivo analysis are in figS7. BMDMs were stained FITC rat anti-mouse/human CD11b (clone M1/70, BioLegend, 1:100) and PE rat anti-mouse F4/80 (clone: BM8, BioLegend, 1:50) and the gating strategies are in figS6.

### CyTOF and recall responses

Single cell suspensions for CyTOF and recall responses were obtained from the lungs of infected mice using the Lung dissociation kit and gentleMACS dissociator, as per the manufacturers recommendations (Miltenyi Biotec). Following lysis of red blood cells, cells were collected by centrifugation, resuspended in PBS and passed through a 40 μm cell strainer. The staining panel is shown in supplementary Table 1. Using the MaxPar conjugation kit, antibodies were conjugated according to the manufacturer’s protocol. Before marker staining, 1 × 10^6^ cells per sample were barcoded with metal-labeled CD45 antibodies in the presence of an Fc blocker for 30 minutes at room temperature, then washed three times with CyFACS buffer (PBS with 0.1% BSA and 2 mM EDTA). The barcoded cells were pooled and stained with a metal-labeled surface stain antibody cocktail for 60 minutes at RT. After washing, the cells were stained for viability using the cisplatin analogue dichloro-(ethylenediamine) palladium (II) in PBS for 10 minutes at RT. After two CyFACS buffer washes and one in PBS, the cells were fixed overnight in 2% formaldehyde solution containing DNA Cell-ID Intercalator-103Rh (Standard BioTools). Prior to acquisition, cells were washed once in CyFACS buffer and twice in water, then diluted to 1 × 10^6^ cells/ml in water containing 15% EQ Four Element Calibration Beads (Standard BioTools) and filtered to avoid clogging of the flow channel. Data acquisition was performed at around 300 cells/s using a Helios mass cytometer (Standard BioTools), with Flow Cytometry Standard files normalized to the EQ bead signal. Initial gating and debarcoding were conducted in CytoBank software (54) and with the *Louvain* algorithm using *FindClusters* in Seurat.

To determine recall responses, single cell lung cell suspensions from infected WT and Clec7a^-/-^ mice were stimulated with purified protein derivative (PPD; obtained from Miyazaki Onsendo), the supernatants were collected after 72 hr, and the IFN-γ level determined using ELISA (BD Bioscience).

### SDS-PAGE and Protein Immunoblot

Cells were washed with 1X DPBS and lysed on ice with RIPA buffer (Millipore, 20-188) containing complete EDTA-free protease inhibitor (ThermoFisher Scientific, 78445). Samples were boiled at 95-100°C for 20 min in LDS (ThermoFisher Scientific, NP008) and NuPage Sample Reducing Agent (ThermoFisher Scientific, NP009) before removal from containment level 3. Subsequently, samples were loaded onto a 4–12% Bis-Tris gel (ThermoFisher Scientific, WG1403BOX) and electrophoresis was performed at 100 V for 120 min. The gels were then transferred onto a PVDF membrane (ThermoFisher

Scientific, IB24002) using an iBlot2 (ThermoFisher Scientific, IB21001) with the P0 program. Following transfer, membranes were blocked in 5% skimmed milk powder in 1X Tris buffer saline with 0.05% Tween-20 (TBS-T) for 1 h at room temperature. After three washes with TBS-T, membranes were incubated with primary antibodies overnight at 4°C. Primary antibodies were collected for reuse the next day, and membranes were washed three times in TBS-T before incubation with horseradish peroxidase (HRP) conjugated secondary antibody for 1 h at room temperature. Membranes were developed using chemiluminescence reagent (Merck, WBLUF0500) and imaged on an Amersham GE Imager 680 (GE Healthcare, U.K.). A molecular weight ladder (Abcam, 116028) was included in all protein immunoblot. Antibodies used were anti-LC3B (Abcam, ab48394, 1:1,000), anti-p62 (Cell Signaling Technologies, #5114, 1:1,000), anti-Dectin-1 Antibody (Cell Signaling Technologies, #23042, 1:1000), anti-actin HRP (Cell Signalling Technologies, #12262, 1:5,000), anti-rabbit HRP (Promega, W4011, 1:10,000). Full data of protein immunoblot are in supplemental data (Fig. S7).

### Microscopy

70,000-80,000 BMDMs were seeded into an olefin-bottomed 96-well plate (Perkin Elmer, 6055302) the day before infection. The next day, BMDMs were infected with N24-GFP at a MOI of 2:1 (mycobacteria:macrophage) for 2 hr at 37°C with 5% CO_2_. Following 2 hr uptake, cells were washed twice with PBS to remove extracellular bacilli and incubated at 37°C with 5% CO_2_ for lysotracker red staining or indirect immunofluorescence analyses. For lysotracker red staining, the media was replaced with media containing 200 nM LysoTracker Green DND-99 (LTR; Invitrogen, L7528) and NucBlue ReadyProbes™ (Invitrogen, R37605). The plates were then incubated for 30 minutes at 37 °C and 5% CO2. Following the incubation, the plates were washed once with 1X DPBS, and fresh media were added. Imaging was done with OPERA Phenix microscope (Perkin Elmer, Germany). The objective used was a 63× 1.4 NA water-immersion objective with a 10% overlap between adjacent fields selected. A single snapshot of each field was taken at the given time point. For detection, λex = 405 nm /λem = 435-480 nm was used for NucBlue, λex = 488 nm/λem = 500-550nm for Mtb N24, and λex = 561 nm/λem = 570–630 nm nm for LysoTracker Red. Detection was done using a 16-bit sCMOS camera. Segmentation and analysis were performed using Harmony software (Perkin Elmer, version 4.9). DAPI signal from a single z-plane was detected using the ‘Find Image Region’ building block, next ‘Find Spots’ building block was used to perform bacterial segmentation. Each individual region of interest was transformed into a mask and extended by using the ‘Find Surrounding Regions’ building block with an individual threshold of 0.8 and including the input region. This mask was used to determine the LysoTracker mean intensity associated to each Mtb region. The mean intensity of LysoTracker associated to every Mtb was calculated and mean of every time point and condition was imported to RStudio (Version 2023.06.1+524). The results were then exported as a .csv file.

For indirect immunofluorescence, cells were fixed in 4% PFA diluted in PBS 48 hr after infection and kept overnight at 4°C. The samples were quenched with 50 mM NH_4_Cl in PBS for 10 min at room temperature and permeabilised with 0.3% Triton X-100, 5% FBS in PBS for 30 min. Antibodies were diluted in PBS containing 5% FBS and incubated for 1 hr at room temperature. Between antibodies, cells were washed three times in PBS. Nuclei were stained for 10 min with DAPI (ThermoFisher, D1306) diluted 1:10000 in PBS. Antibodies used were anti-LAMP2 (Development Studies Hybridoma Bank, ABL-93), anti-p62 (abcam, EPR4844), anti-LC3B (MBL, PM036) and anti-rabbit-Cy5 (Life Technologies, A10523) or anti-rat Cy5 or Cy3 (alpha Diagnostic International). Samples were imaged on a 63× 1.15NA water immersion objective. Images were acquired in confocal mode, a binning of 1. DAPI was detected using λex = 405 nm/λem = 435-480 nm, GFP was detected using λex = 488 nm /λem = 500-550nm and Cy5 was detected using λex = 640 nm /λem = 650-760 nm. Fluorescence was detected using a 16 bits scMOS camera. Segmentation was performed using the Harmony software (Perkin Elmer, version 4.9) where the DAPI signal from a single z-planes was detected using the ‘Find Nuclei’ building block, next ‘Find Spots’ building block was used to perform bacterial segmentation. Following the segmentation, bacteria were filtered based on size and intensity in the GFP channel to avoid the segmentation of non-bacterial objects. Next, the intensity of Cy5 was calculated on each bacterial object using the mask generated by ‘Find Spot’. A threshold was established using positive bacteria and based on the intensity difference, the percentage positive events were calculated with the filtered bacterial objects. Figures were compiled using Adobe Illustrator 2020 V24.3 (Adobe Inc. USA). For all imaging experiments, 40 fields of view were imaged and analysed in each of 3 separate wells for every experiment, involving an analysis of at least 700 cells in each well.

### Ligand isolation and identification

For ligand purification, mycobacteria were collected by centrifugation, resuspended in double distilled water (DDW), autoclaved and lyophilized. To extract hydrophilic components, dried mycobacteria were added to DDW and boiled at 100ºC for 1 hr. After centrifugation (4°C, 9080 *g*, 30 minutes), the supernatant was filtered (0.22 µm filter, SARUTORIUS) and concentrated by freeze-drying. After lyophilization, DDW was added to the dried components to a final concentration of 100 mg/ml (the hot water extract - HWE). This extract was added to 85% (v/v) ethanol and centrifuged (4°C, 9080 *g*, 1 hr) to precipitate polysaccharides. The precipitate was suspended by DDW, ultrafiltered (Vivaspin 20, SARUTORIUS) and then the concentrated components were divided into 20 fractions using AEC (HiTrap HP, Cytiva). Next, the flow-through fractions were combined and separated using gel filtration column chromatography (Superdex^™^ 200 10/300 column, Cytiva), and saccharides were detected using the phenol-sulfuric acid method by measuring the absorption at 492 nm.

To obtain hydrophobic components from *M. tuberculosis* H37Rv, dried bacteria was dissolved in chloroform-methanol (2:1) (10 mg/ml) and agitated by rotation for 2 hours. After centrifugation (RT, 1500 *g*, 10 minutes), the supernatant was filtered and evaporated by nitrogen gas and adjusted at 10 mg/ml in chloroform-methanol (2:1).

For glycogen measurements, a starch assay kit (Cell Biolabs, Inc.) was used. For iodine staining of bacterial colonies, 5 mM potassium Iodine reagent (Nacali-tesc) was added to bacterial culture plates, incubated in the dark for 30 min, washed gently by PBS and then visualized. For iodine staining of alpha-glucans, 100 µl of 5 mM potassium iodine reagent was added to 100 µl of each component to be tested, as indicated, incubated for 10 minutes in the dark and then visualized.

For NMR, 10 mg of the purified ligand was dissolved with 600 μL of D_2_O (99.9%) for NMR measurements. The 1D ^1^H, 2D, and ^1^H-^1^H CLIP-COSY experiments for the ligand were performed on Bruker Avance III 800 MHz spectrometer equipped with a cryogenic probe at 298K of sample temperature. The 1D ^1^H NMR spectral data were collected with 32K data points of FID in 14 ppm of spectral width by 4 scanning. The 2D ^1^H-^1^H CLIP-COSY spectral data were collected with 4K data points in the F2 dimension and 1K increments by 16 scanning in States-TPPI quadrature detection in the F1 dimension, and the spectral width in both dimensions were 14 rpm with 4.7 ppm of offsets. The inter-scan delays of those NMR experiments were 2 sec. Those 2D NMR data were processed and analyzed with the software NMRPipe/NMRDraw (55) and NMRFAM-Sparky (56), respectively. The 1D saturation transfer difference (STD) ^1^H NMR experiments were performed on Bruker Avance III 950 MHz spectrometer equipped with a cryogenic probe at 298K of sample temperature. The ^1^H STD spectral data were collected with 40K data points of the FID in 12 ppm spectral width by continuously applying 50 msec of Gaussian-shaped radio frequency pulses during 2.5 sec to selectively irradiate 0.426 and -100 ppm ^1^H regions to achieve on- and off-resonance saturations of methyl groups of the protein, respectively, by 5760 scanning in an interleaving manner. The 1D NMR data were processed and analyzed with the software TopSpin 3.6.2 (Bruker).

### Statistics

All experiments we performed independently at least twice, unless otherwise indicated. All statistics analysis were conducted in GraphPad Prism 10.2.3 and two-sided testing was used. When ANOVA was used to analyse multiple results, p-values were adjusted using Tukey’s multiple comparisons test. In all graphs, p-values < 0.05 are represented by an asterisk.

## Supplementary Material

supplemental

## Data Availability

All data are available in the main text or the supplementary materials. Tabulated data underlying Figs. 1 to 5 and figs. S1 to S5 are provided in Suppl. Excel_seq1_v2. All data are available in the main text or the supplementary materials. Mice, mycobacterial strains, and other unique reagents generated in this study may be available from the corresponding author under a material transfer agreement, subject to institutional and biosafety regulations.

## One-sentence summary

Mycobacterial α-glucans target Dectin-1, altering phagosomal maturation and enhancing intracellular bacterial survival.

## References

[1] Chai Q, Wang L, Liu CH, Ge B (2020). New insights into the evasion of host innate immunity by Mycobacterium tuberculosis. Cell Mol Immunol.

[2] Chandra P, Grigsby SJ, Philips JA (2022). Immune evasion and provocation by Mycobacterium tuberculosis. Nat Rev Microbiol.

[3] Brown GD, Willment JA, Whitehead L (2018). C-type lectins in immunity and homeostasis. Nat Rev Immunol.

[4] Cheng SC (2014). mTOR- and HIF-1alpha-mediated aerobic glycolysis as metabolic basis for trained immunity. Science.

[5] Ulland TK (2017). TREM2 Maintains Microglial Metabolic Fitness in Alzheimer’s Disease. Cell.

[6] Wang S (2022). TREM2 drives microglia response to amyloid-beta via SYK-dependent and -independent pathways. Cell.

[7] Yin L (2024). Oroxylin A-induced Trained Immunity Promotes LC3-associated Phagocytosis in Macrophage in Protecting Mice Against Sepsis. Inflammation.

[8] Wagener M, Hoving JC, Ndlovu H, Marakalala MJ (2018). Dectin-1-Syk-CARD9 Signaling Pathway in TB Immunity. Front Immunol.

[9] Yadav M, Schorey JS (2006). The beta-glucan receptor dectin-1 functions together with TLR2 to mediate macrophage activation by mycobacteria. Blood.

[10] Rothfuchs AG (2007). Dectin-1 interaction with Mycobacterium tuberculosis leads to enhanced IL-12p40 production by splenic dendritic cells. J Immunol.

[11] Shin DM (2008). Mycobacterium abscessus activates the macrophage innate immune response via a physical and functional interaction between TLR2 and dectin-1. Cell Microbiol.

[12] van de Veerdonk FL (2010). Mycobacterium tuberculosis induces IL-17A responses through TLR4 and dectin-1 and is critically dependent on endogenous IL-1. J Leukoc Biol.

[13] Zenaro E, Donini M, Dusi S (2009). Induction of Th1/Th17 immune response by Mycobacterium tuberculosis: role of dectin-1, Mannose Receptor, and DC-SIGN. J Leukoc Biol.

[14] Branzk N (2014). Neutrophils sense microbe size and selectively release neutrophil extracellular traps in response to large pathogens. Nat Immunol.

[15] Romero MM (2016). Reactive oxygen species production by human dendritic cells involves TLR2 and dectin-1 and is essential for efficient immune response against Mycobacteria. Cell Microbiol.

[16] Das R (2013). Macrophage migration inhibitory factor (MIF) is a critical mediator of the innate immune response to Mycobacterium tuberculosis. Proc Natl Acad Sci U S A.

[17] Marakalala MJ (2011). The Syk/CARD9-coupled receptor Dectin-1 is not required for host resistance to Mycobacterium tuberculosis in mice. Microbes and Infection.

[18] Lerner TR (2020). Mycobacterium tuberculosis cords within lymphatic endothelial cells to evade host immunity. JCI Insight.

[19] Mourik BC (2019). Mycobacterium tuberculosis clinical isolates of the Beijing and East-African Indian lineage induce fundamentally different host responses in mice compared to H37Rv. Sci Rep.

[20] Moreira-Teixeira L (2020). Mouse transcriptome reveals potential signatures of protection and pathogenesis in human tuberculosis. Nat Immunol.

[21] Queval CJ, Brosch R, Simeone R (2017). The Macrophage: A Disputed Fortress in the Battle against Mycobacterium tuberculosis. Front Microbiol.

[22] Brown GD (2003). Dectin-1 mediates the biological effects of beta-glucan. J Exp Med.

[23] Gutierrez MG (2004). Autophagy is a defense mechanism inhibiting BCG and Mycobacterium tuberculosis survival in infected macrophages. Cell.

[24] Queval CJ (2017). Mycobacterium tuberculosis Controls Phagosomal Acidification by Targeting CISH-Mediated Signaling. Cell Rep.

[25] Bernard EM (2020). M. tuberculosis infection of human iPSC-derived macrophages reveals complex membrane dynamics during xenophagy evasion. J Cell Sci.

[26] Golovkine GR (2023). Autophagy restricts Mycobacterium tuberculosis during acute infection in mice. Nat Microbiol.

[27] Aylan B (2023). ATG7 and ATG14 restrict cytosolic and phagosomal Mycobacterium tuberculosis replication in human macrophages. Nat Microbiol.

[28] Takano T (2017). Dectin-1 intracellular domain determines species-specific ligand spectrum by modulating receptor sensitivity. J Biol Chem.

[29] Adachi Y (2004). Characterization of beta-Glucan Recognition Site on C-Type Lectin, Dectin 1. Infect Immun.

[30] Tone K, Stappers MHT, Willment JA, Brown GD (2019). C-type lectin receptors of the Dectin-1 cluster: Physiological roles and involvement in disease. Eur J Immunol.

[31] van de Weerd R (2015). A murine monoclonal antibody to glycogen: characterization of epitope-fine specificity by saturation transfer difference (STD) NMR spectroscopy and its use in mycobacterial capsular alpha-glucan research. Chembiochem.

[32] Rashid AM (2016). Assembly of alpha-Glucan by GlgE and GlgB in Mycobacteria and Streptomycetes. Biochemistry.

[33] Sambou T (2008). Capsular glucan and intracellular glycogen of Mycobacterium tuberculosis: biosynthesis and impact on the persistence in mice. Mol Microbiol.

[34] Bussi C, Gutierrez MG (2019). Mycobacterium tuberculosis infection of host cells in space and time. FEMS Microbiol Rev.

[35] Chen D, Fearns A, Gutierrez MG (2025). Mycobacterium tuberculosis phagosome Ca(2+) leakage triggers multimembrane ATG8/LC3 lipidation to restrict damage in human macrophages. Sci Adv.

[36] Tam JM (2014). Dectin-1-dependent LC3 recruitment to phagosomes enhances fungicidal activity in macrophages. J Infect Dis.

[37] Deleyto-Seldas N, Efeyan A (2021). The mTOR-Autophagy Axis and the Control of Metabolism. Front Cell Dev Biol.

[38] Pagan AJ (2022). mTOR-regulated mitochondrial metabolism limits mycobacterium-induced cytotoxicity. Cell.

[39] Petit J (2019). Studies Into beta-Glucan Recognition in Fish Suggests a Key Role for the C-Type Lectin Pathway. Front Immunol.

[40] Rogers H, Williams DW, Feng GJ, Lewis MA, Wei XQ (2013). Role of bacterial lipopolysaccharide in enhancing host immune response to Candida albicans. Clin Dev Immunol.

[41] Kimmey JM (2015). Unique role for ATG5 in neutrophil-mediated immunopathology during M. tuberculosis infection. Nature.

[42] Houben D (2012). ESX-1-mediated translocation to the cytosol controls virulence of mycobacteria. Cell Microbiol.

[43] Geurtsen J (2009). Identification of mycobacterial alpha-glucan as a novel ligand for DC-SIGN: involvement of mycobacterial capsular polysaccharides in host immune modulation. J Immunol.

[44] Kalscheuer R (2019). The Mycobacterium tuberculosis capsule: a cell structure with key implications in pathogenesis. Biochem J.

[45] Prados-Rosales R (2016). The Type of Growth Medium Affects the Presence of a Mycobacterial Capsule and Is Associated With Differences in Protective Efficacy of BCG Vaccination Against Mycobacterium tuberculosis. J Infect Dis.

[46] Palma AS (2015). Unravelling glucan recognition systems by glycome microarrays using the designer approach and mass spectrometry. Mol Cell Proteomics.

[47] Xisto M (2023). An Alpha-Glucan from Lomentospora prolificans Mediates Fungal-Host Interaction Signaling through Dectin-1 and Mincle. J Fungi (Basel).

[48] Adams EL (2008). Differential high affinity interaction of Dectin-1 with natural or synthetic glucans is dependent upon primary structure and is influenced by polymer chain length and side chain branching. J Pharmacol Exp Ther.

[49] Restrepo BI (2021). Human monocyte-derived macrophage responses to M. tuberculosis differ by the host’s tuberculosis, diabetes or obesity status, and are enhanced by rapamycin. Tuberculosis (Edinb).

[50] Ferwerda B (2009). Human dectin-1 deficiency and mucocutaneous fungal infections. N Engl J Med.

[51] Brites D, Gagneux S (2015). Co-evolution of Mycobacterium tuberculosis and Homo sapiens. Immunol Rev.

[52] Taylor PR (2007). Dectin-1 is required for beta-glucan recognition and control of fungal infection. Nat Immunol.

[53] Miyamoto Y (2010). Novel rhamnosyltransferase involved in biosynthesis of serovar 4-specific glycopeptidolipid from Mycobacterium avium complex. J Bacteriol.

[54] Nowicka M (2017). CyTOF workflow: differential discovery in high-throughput high-dimensional cytometry datasets. F1000Res.

[55] Delaglio F (1995). NMRPipe: a multidimensional spectral processing system based on UNIX pipes. J Biomol NMR.

[56] Lee W, Tonelli M, Markley JL (2015). NMRFAM-SPARKY: enhanced software for biomolecular NMR spectroscopy. Bioinformatics.

