# Supplementary material for: Mycobacterial α-glucans hijack Dectin-1 to facilitate intracellular bacterial survival
Source: Sci Immunol. Author manuscript; Available in PMC 2026 Jan 29. (PMC7618678; doi:10.1126/sciimmunol.adw0732)
Supplement: supplemental [file EMS212109-supplement-supplemental_.docx]

**List of Supplementary Materials**

Figure S1 Dectin-1 deficient mice are resistant to mycobacterial infection

Figure S2 Dectin-1 promotes intracellular mycobacterial survival

Figure S3 Dectin-1 recognizes mycobacterial α-glucan

Figure S4 Avirulent mycobacteria also express the Dectin-1 ligand

Figure S5 Differences in alpha-glucan between *M. smegmatis* wild-type and Δ*glgB*

Figure S6 Establishment of C57BL6/J Dectin-1-deficient mice using CRISPR/CAS9

Table S1 Antibodies list for CyTOF

**Supplementary figure legends**

**Supplementary figure 1: Dectin-1 deficient mice are resistant to mycobacterial infection**

(**A**) Survival of wild type and *Clec7a*^–/–^ mice after i.n. infection with 600CFU of MTB Beijing (129Sv mice), 1300CFU MTB Erdman (C57BL6/J mice) or 700CFU H37RV (129Sv mice), as indicated. (**B**) Pulmonary H&E staining of 129Sv wild type and *Clec7a^–/–^* mice 3 weeks after infection (top) with 628 CFU MTB N24. Scale bar = 100µm, as indicated. Arrow indicates areas of cell infiltration in infected lungs. (**C**) UMAP analysis of cells isolated 4 weeks after infection with MTB Erdman C57BL6/J lung tissues using CyTOF. Cells were grouped into nine clusters according to classic lineage marker expression. (**D**) Heatmap showing surface marker expression for the annotation of immune cells (**E**) Pulmonary cell numbers in the lungs of uninfected or 4 weeks post MTB Erdman infection (100CFU) C57BL/6 WT or Clec7a^–/–^ mice measured through CyTOF analyses. Data is from a single experiment (n= 4 mice per group). (**F**) Total (left) and number of GFP positive neutrophils (right) in the lungs of mice 14 days after i.n. infection with 400-600 CFU of MTB N24-GFP (n = 18). Data shown are pooled data from two independent experiments. (**G**) Pulmonary cells isolated from MTB Erdman infected (100CFU) C57BL/6 WT or *Clec7a*^–/–^ mice at 4 weeks post infection were left unstimulated or stimulated with PPD (1 µg/ml) for 72 hr and IFN-γ levels in the supernatant were measured by ELISA (single experiment performed on samples isolated from five mice). Log-rank tests (**A**), ANOVA (**E**) and Student’s t-tests (**F**, **G, J**) were used for statistical analyses. **p* < 0.05.

**Supplementary figure 2: Dectin-1 promotes intracellular mycobacterial survival**

(**A**) CFU counts at day 0 (loading control) and day 6, as indicated, following infection of C57BL/6J wild type and *Clec7a*^–/–^ BMDMs with MTB Erdman, in the presence or absence of 10ng/ml IFNγ, as indicated. Data show mean ± SD of one representative experiment performed in triplicate. (**B**) Supernatant TNFα levels 24 hr after infection of C57BL6/J wild type and *Clec7a*^–/–^ BMDMs with MTB Erdman. Data show mean ± SD of one representative experiment performed in triplicate. (**C**) CFU counts at day 0 (loading control) and day 6, as indicated, following infection of 129Sv wild type and *Clec7a*^–/–^ BMDMs with MTB Beijing. Data show mean ± SD of a single experiment in triplicate. (**D**) Confocal microscopy images (left) and quantitation (right) of Lysotracker Red (red) localization in 129Sv wild type and *Clec7a*^–/–^ BMDMs 24hr following infection with MTB N24-GFP. DNA was stained with DAPI (blue). Scale bar = 10µm. Data show mean ± SD . (**E**) Confocal microscopy images (left) and quantitation (right) of p62 (red) localization in 129Sv wild type and *Clec7a*^–/–^ BMDMs 24hr following infection with MTB N24-GFP. DNA was stained with DAPI (blue). Scale bar = 10µm. Data show mean ± SD. (**D**, **E**) Data representative of 2 experimental repeats with least 25 fields of view analyzed in each experiment, with three technical repeats conducted in each experiment. (**F**) Western blot analysis of autophagy markers, p62 and LC3, following infection of 129Sv wild type and *Clec7a*^–/–^ BMDMs with MTB N24 at the time points indicated. Uninfected (uninf) samples shown as controls. (**G**) Survival of MTB N24 following culture with rapamycin (0.1, 1 and 10 µM) for 24hrs. Isoniazid (0.1 µg/ml; INH) was included as a positive control (pooled data from two experiments [n=6 per experiment]). Student’s *t*-test (A-**E**) or ANOVA (**G**) were used for statistical analyses. **p* < 0.05**.**

**Supplementary figure 3: Dectin-1 recognizes mycobacterial α-glucan**

(**A**) NFAT-GFP reporter cells expressing human Dectin-1 were stimulated with hot water extracts (HWE) (0, 10, 30 and 100 µg/mL) isolated from MTB N24 for 18 hr. GFP expression was analyzed using flow cytometry. Unstimulated cells are shown as a control (-). (**B**) NFAT-GFP reporter cells expressing human Dectin-1 were stimulated with chloroform methanol (CM) or hot water extracts (HWE; 0.01, 0.03, 0.1, 0.3, and 1 mg/mL) isolated from MTB H37Rv for 18hr. GFP expression was analyzed using flow cytometry. Unstimulated cells (-) are shown as a control. (**C**) NFAT-GFP reporter cells expressing human Dectin-1 were stimulated with ultrafiltration-purified hydrophilic components (0, 0.1, 0.3, 1 mg/ml) from liquid media and culture media, as indicated. (**D**) Graphical representation of purification protocol to isolate the MTB Dectin-1 ligand. (**E**) Ultrafiltrated MTB components were separated using AEC (anion exchange column) into 20 fractions. The absorption of each fraction was detected at 492nm for saccharides using the phenol-sulfuric acid method. Reporter cells expressing human Dectin-1 were stimulated with the fractions indicated for 18hr and analyzed for GFP expression by flow cytometry. (**F**) (top) AEC fractions were further separated using gel column chromatography and the absorption of each fraction was detected at 492 nm for saccharide using the phenol-sulfuric acid method. Mycobacterial fractions were tested for activity by stimulating reporter cells expressing human Dectin-1 for 18hr and analyzing for GFP expression by flow cytometry. (bottom, colored circles) Separation of various sized dextran’s were used as molecular markers. (**G**) NFAT-GFP reporter cells expressing murine Dectin-1 and CTLD mutants, as indicated, were stimulated with HWE derived from MTB H37Rv. (**H**) Direct binding assay of recombinant Fc-Dectin-1 protein (10 µg/ml) to mycobacterial components coated on a nitrocellulose membrane. A related recombinant Fc protein (Fc-CLEC12A) was used as a control (Fc-cntrl) (10 µg/ml). (**I**) Analysis of the α-glucan structure using iodine stain of amylose (1 mg/ml), amylopectin (1 mg/ml), bovine glycogen (10 mg/ml), and purified components in gel fractions (10 mg/ml). (**J**) The percentage of α-glucan in purified fractions, as indicated, were determined using a starch measurement kit. (**K**) Reporter cells expressing human Dectin-1 were stimulated with HWE treated with various glycosidases, as indicated, for 16-20hr and analyzed for GFP expression by flow cytometry. (**L**) NFAT-GFP reporter cells expressing various CLRs, as indicated, murine Dectin-1 were stimulated with purified α-glucan (100 µg/ml) for 18hr. GFP expression was analyzed using flow cytometry. (**M**) ^1^H-NMR spectrum (800 MHz, D_2_O(99.9%)) of purified Dectin-1 ligand for assignment: (Left) the ^1^H NMR resonances of the ligand were assigned and denoted on the 1D ^1^H NMR spectrum as shown upper the 2D ^1^H-^1^H CLIP-COSY NMR spectrum. The magenta arrows denoted on the 2D ^1^H-^1^H CLIP-COSY NMR spectrum indicate *J*^3^-coupled ^1^H-^1^H NMR resonance correlations in a sequential assignment walking manner. (Right) The ^1^H NMR resonances of reduced-end moiety of the ligand were assigned and denoted on the 1D ^1^H NMR spectrum as shown upper the 2D ^1^H-^1^H CLIP-COSY NMR spectrum. The magenta arrows denoted on the 2D ^1^H-^1^H CLIP-COSY NMR spectrum indicate *J*^3^-coupled ^1^H-^1^H NMR resonance correlations in a sequential assignment walking manner. (**N**) Analysis of Dectin-1 binding to purified ligand by STD-NMR. The ^1^H STD NMR experiments using a sample containing ligands and proteins were performed by irradiating radio frequency pulses to the ^1^H resonances of methyl groups of the proteins in an interleaving manner. The on- or off-resonance as denoted on the upper panel indicate that red- and blue-colored ^1^H NMR spectra were measured with and without saturating the ^1^H resonances of methyl groups of the proteins. The ^1^H NMR spectrum in the lower panel is differential spectrum generated by subtracting the ^1^H NMR spectra of on-resonance (spectrum B, colored by red in the upper panel) from that of the off-resonances (spectrum A, colored by blue in the upper panel). (**G, L**) Data are the mean ± SD of duplicate or (**A-C, J, K**) triplicate assays and representative of at least two independent experiments. (**E, F, H, I**) Data is single assay and representative of at least two independent experiments. ANOVA (**A-C, J**) or Student’s *t*-tests (**K, L**) were used for statistical analyses. **p*< 0.05.

**Supplementary figure 4: Avirulent mycobacteria also express the Dectin-1 ligand**

(**A**) CFU counts at day 6 following infection of 129Sv wild type and *Clec7a*^–/–^ BMDMs with *M. bovis* BCG, harvested from liquid media or directly from agar plates. (**B**) ^1^H-NMR spectrum of purified mycobacterial ligands from *M. smegmatis* or *M. bovis* BCG, as indicated. (**C**) CFU counts at day 0 (loading control) following infection of 129Sv wild type and *Clec7a*^–/–^ BMDMs with *M. smegmatis* and the Δ*glgB* mutant strain, harvested directly from agar plates. (**D**) CFU counts at day 3 and day 6 following infection of 129Sv wild type and *Clec7a*^–/–^ BMDMs with *M. smegmatis*, in the presence or absence of 1µM rapamycin, as indicated. (**E**) CFU counts at day 6 following infection of 129Sv wild type BMDMs with *M. smegmatis*, in the presence or absence of 1µM torin-1, as indicated. (**A**, **C**, **D**, **E**) Pooled data from two independent experiments showing mean ± SD. ANOVA (**C**, **D**) or Student’s *t*-test (A, **E**) were used for statistical analyses. **p* < 0.05.

**Supplementary figure 5: Differences in alpha-glucan between *M. smegmatis* wild-type and Δ*glgB***

(**A**) Iodine staining of bacterial colonies of *M. smegmatis* wild-type and Δ*glgB* by 5 mM potassium Iodine reagent. (**B**) CFU counts 8hr after infection of 129Sv wild type and *Clec7a*^–/–^ mice with *M. smegmatis* wild type (wt smeg) or the Δ*glgB* mutant, as indicated. Data from one representative t experiment showing mean ± SD. (**C**) Iodine staining of bacterial colony of *M. smegmatis* Δ*glgB* and the *glgB* complemented strain with 5 mM potassium Iodine reagent. (**D**) CFU counts at day 2 following infection of C57BL6/J wild type and *Clec7a*^–/–^ BMDMs with *M. smegmatis* wild type (wt smeg), the Δ*glgB* mutant, or the *glgB* complemented strains, as indicated. Data are the mean ± SD from a representative experiment. ANOVA (B, D) used for statistical analyses. **p*< 0.05.

**Supplementary figure 6: Establishment of C57BL6/J Dectin-1-deficient mice using CRISPR/CAS9**

(**A**) Sequence of exon 1 and intron in the *Clec7a* gene locus. Capital letters indicate exon, and small letters indicate the intron of the *Clec7a* gene. One mutated allele (Δ5) lost 5 nucleotides in exon 1, and another mutated allele (Δ44) lost 44 nucleotides in the gene (19 nucleotides of exon 1 was deleted). (**B**) Heteroduplex mobility assay for genotyping. Primers for PCR are indicated as arrows in (**A**). (**C**) Analysis of Dectin-1 expression on BMDMs from C57BL6/J wild type (WT) and *Clec7a*^–/–^ mice. Cells were stained with isotype control and anti-Dectin-1 monoclonal antibody (clone 2A11). (**D**) Flow cytometric analysis of expression of murine Dectin-1 and mutants, as indicated, on the surface of the NFAT-GFP reporter cells. White histograms indicate isotype control staining.

**Supplementary figure 7: Gating strategies of in vivo analysis**

(A) Analysis of neutrophils, alveolar macrophages, and interstitial macrophages in Figure 1B and 1C. (B) Analysis of GFP-positive cells in alveolar macrophages and neutrophils in Figure 1C and figure S1F.

**Table S1**

Antibodies list for CyTOF
